# Sperm quality and *in vitro* fertilizing ability of boar spermatozoa stored at 4 °C versus conventional storage for 1 week

**DOI:** 10.3389/fvets.2024.1444550

**Published:** 2024-09-13

**Authors:** Ida Hallberg, Jane M. Morrell, Pack Malaluang, Anders Johannisson, Ylva Sjunnesson, Denise Laskowski

**Affiliations:** ^1^Department of Clinical Sciences, Centre for Reproductive Biology in Uppsala (CRU), Swedish University of Agricultural Sciences, Uppsala, Sweden; ^2^Department of Animal Biosciences, Swedish University of Agricultural Sciences, Uppsala, Sweden

**Keywords:** porcine, *in vitro* embryo production, semen storage, artificial insemination, long term boar semen storage

## Abstract

**Introduction:**

Since boar spermatozoa show a marked deterioration in sperm quality when cooled, insemination doses are usually stored at 16–18 °C. However, maintaining this temperature during transport of semen doses is challenging, particularly during the summer months. An alternative could be to store the doses at 4 °C if cold-shock to the sperm could be prevented. The objective of this study was to evaluate boar sperm quality and fertility in *in vitro* fertilization after storage in AndroStar Premium at 4 °C for 1 week.

**Methods:**

Insemination doses (*n* = 9) in AndroStar Premium from a commercial boar semen collection station were transported to the laboratory at approximately 20 °C. At the laboratory, sperm quality evaluation and was preformed and each dose was split; half of each ejaculate was stored in a climate-controlled box at 16–18 °C, the other was slowly cooled to 4 °C. Both samples were stored for 1 week before further sperm quality evaluation and *in vitro* fertilization (IVF) were performed. Mean values were tested using generalized linear regression, with treatment and boar as fixed factors; *p* ≤ 0.05 was considered significant.

**Results:**

Sperm membrane integrity (mean ± sem: 91 ± 0.05 and 83 ± 0.09% for 16 and 4 °C, respectively) and superoxide production (6.79 ± 2.37 and 13.54 ± 6.23% for 16 and 4 °C, respectively), were different between treatments. The DNA fragmentation index was lower in cold-stored samples than in conventionally stored samples (3.74 ± 2.25 and 7.40 ± 3.36% for 4 and 16 °C, respectively). The numbers of oocytes developing to blastocyst on Day 6 (mean ± sd: 9.0 ± 8.0 and 6.0 ± 5.0%, for storage at 16 and 4 °C, respectively) were not different between treatments.

**Discussion:**

Therefore, storage of boar semen doses in AndroStar Premium at 4 °C for up to 7 days would be a viable alternative to current praxis.

## 1 Introduction

One of the major challenges in porcine reproductive biotechnologies lies in maintaining boar sperm quality in semen doses destined for artificial insemination (AI). This reproductive biotechnology is considered to be the most frequently used in pig breeding (1). Almost all pig breeding involves AI (2), enabling better use of genetics, improved disease control and increased biosecurity than natural mating (3). Furthermore, AI improves the farm economy, being cheaper than natural mating while providing similar reproductive efficiency, and permitting access to a wider range of sires than is possible if farmers keep their own boars (4, 5).

In contrast to the situation in cattle AI, liquid semen, rather than frozen semen, is preferred for the vast majority of pig AIs (6), since there is considerable variation in the success of cryopreservation among boars and even among ejaculates from the same boar (7). The low ratio of cholesterol to phospholipid in the boar sperm membrane is believed to be one of the contributing factors in making boar sperm particularly susceptible to the adverse effects of cryopreservation (8). Boar spermatozoa are reported to be sensitive to cooling below 16 °C, with temperature-induced lipid phase changes to the sperm membranes that have a profound effect on sperm survival and functionality (9). However, results are conflicting: in one study, acceptable fertility rates could be achieved in Androhep extender under storage conditions as low as 12 °C for 48 h (10). In contrast, storage temperatures below 20 °C affected sperm motility and acrosome integrity in semen from Norwegian Landrace boars (11), although there was considerable variation among boars in sperm survival during storage at 10 °C.

Apart from the deleterious effects of low storage temperatures on boar spermatozoa, temperatures above 17 °C during transport are also deleterious to sperm quality, even when the sperm doses are placed in a sealed, insulated box. Sperm motility of semen doses was impaired during transport at a high ambient temperature of 37 °C and did not recover during subsequent storage at 17 °C (12). Release of heat shock proteins and increased apoptosis were observed, which the authors attributed to activation of adenosine monophosphate (AMP)-activated protein kinase.

Most boar semen for AI is currently stored at 16–18 °C and used within 3–5 days of collection (3). Due to the temperate climate in Sweden, boar semen doses are transported from the boar station to the pig farms at ambient temperature, and are subsequently stored at 16–18 °C. In other countries, where ambient temperatures can be considerably hotter than in Scandinavia, it is difficult to maintain sperm quality during transport. In any case, even when stored at 16–18 °C there is an increase in DNA fragmentation in boar semen after storage, either for approximately 48 h (13) or 72 h (14, 15); the underlying reason for this increase in DNA fragmentation is not known.

Adenosine triphosphate (ATP) production in boar sperm samples is reduced at temperatures below 17 °C, but not all spermatozoa are affected equally (16). This finding indicates that at least some spermatozoa could survive low-temperature storage. The manufacturers of boar semen extenders have developed formulations that they hope could be used to store boar semen at 4 °C. One such commercial extender is AndroStar Premium (Minitube International, Tiefenbach, Germany). Some preliminary studies investigated sperm quality and fertility of boar semen in these extenders [e.g., (17, 18)]. The manufacturer presents data on sperm motility after 9 days of storage in this new extender. If sperm fertility is not compromised by storage at 4 °C, the availability of this extender could be of considerable interest for pig producers.

The objective of this study was to investigate the *in vitro* fertility and sperm quality of boar spermatozoa stored at 4 °C for 1 week compared to conventional storage at 16–18 °C.

## 2 Materials and methods

### 2.1 Experimental design

Ejaculates from nine boars at a commercial boar station were split, with one portion being stored at 16–18 °C (conventional storage) and the other portion at 4 °C for 1 week. Sperm membrane integrity, chromatin integrity and reactive oxygen species production (ROS) were analysed immediately on arrival at the laboratory, and again after storage for 6 or 7 days, at which time the *in vitro* fertilizing capacity was also evaluated. Although the majority of the sperm samples were stored for 7 days, it was necessary to store some for only 6 days for practical reasons. Therefore, on a subsequent occasion, semen samples were used after both day 6 and day 7 of storage, for comparison.

### 2.2 Sperm samples

Ejaculates were collected from nine Hampshire boars at a commercial boar station (Köttforetagen, Hållsta, Sweden) using the gloved hand method and were extended in AndroStar Premium (kind gift of Minitube International GmbH, Tiefenbach, Germany) to give conventional semen doses of 2.4 × 10^9^ spermatozoa in 80 mL. These doses were transported to the Swedish University of Agricultural Sciences in an insulated box at ambient temperature, arriving approximately 4 h after semen collection. After removing 1 mL of sample for sperm quality analyses, half of each sample was stored at 16–18 °C in a climate-controlled box (Unitron, Tørring, Denmark). The other half was slowly cooled to 4 °C, by placing a 50-mL tube containing half the insemination dose in an insulated box inside another insulated box in the cold room at 4 °C. The temperature of the semen doses reached 4 °C after approximately 6 h. Sperm quality was evaluated on arrival at the laboratory and again after 6 or 7 days, when the sperm samples were also used for *in vitro* fertilization (IVF) following standard protocols (19).

### 2.3 Sperm concentration

Sperm concentration was measured with a Nucleocounter SP-100 (Chemometec, Allerød, Denmark). Briefly, 50 μL of semen were mixed with 5 mL Reagent S100 (Chemometec, Allerød, Denmark) to break down cell membranes before loading a cassette containing propidium iodide (PI) with the sperm mixture. The cassette was then placed in the fluorescence reader and sperm concentration was displayed after approximately 30 s.

### 2.4 Flow cytometry

Analyses were performed using a FACSVerse flow cytometer (BDBiosciences; Franklin Lakes, NJ, United States) equipped with standard optics. Sperm samples were first diluted to 2 × 10^6^ spermatozoa/mL with modified Beltsville Thawing Solution (BTS) (20), consisting of: glucose (3.7 g), tri-sodium citrate (0.6 g), sodium hydrogen carbonate (0.13 g), sodium EDTA (0.13 g) and potassium chloride (0.08 g) added to 100 mL distilled water, i.e., the modification was that no antibiotics were added to the mixture.

#### 2.4.1 Membrane integrity

Membrane integrity was evaluated with SYBR-14 and propidium iodide (PI) (Live-Dead Sperm Viability Kit L-7011; Invitrogen, Eugene, OR, United States). The sperm samples, diluted to 2 × 10^6^ spermatozoa/mL in BTS, were stained with 0.6 μL of SYBR-14 (1:50 in BTS; 40 nM) and 3 μL of PI (24 μM). The tubes were kept in the dark at 38 °C for 10 min. After excitation with a blue laser, green fluorescence (FL1) from SYBR-14 was detected with band-pass filter (527/32 nm), while red fluorescence (FL3) from PI was measured using a band-pass filter of 700/54 nm. From each sample, measurements from 50,000 events were collected and quantified as percentages. For the purposes of this experiment, spermatozoa were classified as having intact membranes (SYBR14+, PI−), or damaged membranes (either dead SYBR−, PI+ or SYBR14+, PI+).

#### 2.4.2 Sperm chromatin structure assay

After mixing aliquots of sperm samples (50 μL) 1:1 with TNE buffer (Tris-sodium chloride-EDTA; 0.15 mol/L NaCl, 0.01 mol/L Tris–HCl, 1 mmol/L EDTA, pH 7.4), the samples were snap-frozen in liquid nitrogen and transferred to a − 80 °C freezer and stored until further analysis. The samples were thawed on crushed ice, aliquots (10 μL) were further diluted with TNE buffer (90 μL) and subjected to partial DNA denaturation *in situ* with a detergent solution (0.2 mL; 0.17% Triton X-100, 0.15 mol/L NaCl, and 0.08 mol/L HCl; pH 1.2). Then they were stained with acridine orange (0.6 mL; 6 μg/mL in 0.1 mol/L citric acid, 0.2 mol/L Na_2_HPO_4_, 1 mmol/L EDTA, 0.15 mol/L NaCl; pH 6.0). The samples were analysed using flow cytometry within 3–5 min. For each sample, at least 10,000 events were analyzed at a speed of 200 cells/s after excitation with a blue laser (488 nm). Both forward scatter (FSC) and side scatter (SSC) were collected. The FL1 (green fluorescence) was collected through a band-pass filter (527/32 nm); FL3 (red fluorescence) was collected using a band-pass filter for wavelengths 700/54 nm. After gating for spermatozoa in the FSC-SSC dot-plot, the DNA Fragmentation Index (%DFI, i.e., the ratio of cells with denatured, single-stranded DNA to total spermatozoa acquired) was calculated for each sample using flow cytometry standard (FCS) Express version 5 (*De Novo* Software, Pasadena, CA, United States). The proportion of high DNA staining samples (HDS) was also recorded.

#### 2.4.3 Assessment of reactive oxygen species

Aliquots (300 μL) were stained with Hoechst 33258 at 0.4 μM (HO; Sigma, Stockholm), 0.4 μM hydroethidine (HE; Invitrogen Molecular Probes, Eugene, OR, United States) and 20 μM dichlorodihydro-fluorescein diacetate (DCFDA; Invitrogen Molecular Probes). The samples were incubated at 37 °C for 30 min before analyzing. Excitation was with a blue laser emitting at 488 nm and a violet laser emitting at 405 nm. Detection of green fluorescence from DCFDA (FL1) was via a band-pass filter (527/32 nm), red fluorescence from HE (FL3) was measured using a band-pass filter (700/54 nm), and blue/green fluorescence from Hoechst 33258 (FL5) was detected via a band-pass filter (528/45 nm). In total, 30,000 sperm-specific events were evaluated. After gating for spermatozoa in the FSC-SSC dotplot, they were classified as living or dead superoxide or H_2_O_2_ negative, and living or dead superoxide or H_2_O_2_ positive.

### 2.5 *In vitro* embryo production

Ovaries from gilts were collected at a local slaughterhouse and transported to the laboratory at the Swedish University of Agricultural Sciences where cumulus oocyte complexes (COCs) were harvested from the follicles and matured *in vitro* according to standardized procedures as previously described (19). In brief, the COCs were divided into two groups at random to minimize variation between treatments. For this study, in total 11 batches (replicates) were used, containing in total 874 oocytes leading to 64 developing to the blastocyst stage.

The procedure detailed in Leclercq et al. (19) was followed for IVF, using commercially available porcine oocyte maturation medium (POM), porcine fertilization medium (PFM), and porcine zygote medium (PZM) purchased from Research Institute for the Functional Peptides, FHK Fujihura Industry Co Ltd., Osaka, Japan. Wash media was used for handling oocytes and embryos outside the incubators and was produced on site (gentamicin sulfate 10 μg/mL, L-glutamine MW 146.14 1 mM, PVA 3 μg/mL in Hepes TCM 199; wash media for aspiration of COCs had, in addition, 20 U/mL heparin added). Media to be used in incubators were equilibrated for at least 2 h in 38.5 °C and 5.5% CO₂ before use.

The POM medium used for the first 22 h of maturation was enriched with follicle stimulating hormone (FSH) @ 0.05 IU/mL (FSH Porcine, OOPA00171, Insight Biotechnology, Middlesex, United Kingdom), luteinizing hormone (LH) @ 0.05 IU/mL (LH Protein, OOPA00173, Insight Biotechnology), and dibutyryl adenosine cyclic monophosphate (dbcAMP) @ 1 mM (dbcAMP, sodium salt, 1,698,950, Biogems, Westlake Village, United States). The following 23 h of maturation were carried out in the absence of LH, FSH or dbcAMP. *In vitro* maturation and *in vitro* fertilization were performed in an incubator with 5.5% CO₂ and 38.5 °C in maximum humidity; for *in vitro* culture, 5.5% O₂ was included in the gaseous mix. After maturation of the oocytes for 45 h in total, *in vitro* fertilization was carried out using sperm samples stored at 4 °C for one group of oocytes and those stored at 16–18 °C for the other group of oocytes. Semen preparation consisted of diluting 0.5 mL of each sperm sample in 4 mL of PFM. After mixing, 2 mL of the sperm dilution was placed on top of 4 mL of low density Porcicoll at room temperature (21). The preparations were then centrifuged for 20 min at 300 x g before removing the supernatant. The sperm pellets were diluted in PFM, the concentration was measured and the sperm concentration was adjusted to 0.6 ×10^6^/mL for IVF. The COCs were randomly distributed between the groups; the group sizes varied between 10 and 13 (50 μL drops under oil) and 35–50 (500 μL wells without oil). Ideally, only wells would have been used, but there was a concern that too few good quality oocytes would be available on any 1 day. Therefore, the alternative (culturing 10 oocytes in 50 μL drops of culture medium) was compared with our standard practice (culturing 30–50 oocytes in 500 μL culture medium in wells). Since the results were consistent for both groups, we were able to use the drop method on days when there were insufficient oocytes for wells.

After 24 h in PFM, presumed zygotes were denuded by gentle pipetting, washed and cultured in PZM under oil for 6 days before fixation and staining. During the culture process, cleavage rate and cleavage rate above 2 cell stage were recorded 48 h post fertilization. Developing blastocysts were evaluated at day 5 post fertilization by light microscopy directly in the wells or drops. On day 6 post fertilization, all oocytes and embryos were collected, fixed in 2% paraformaldehyde and stained using 4′,6-diamidino-2-phenylindole (DAPI) to determine the number of spermatozoa still firmly attached to the zona pellucida. The number of blastocysts and developmental stages were documented. Note that the number of spermatozoa still attached to the zona pellucida at this stage might not correspond to the number that were attached in connection with fertilization, especially since the presumptive zygotes had been washed, but it still provides an indication of whether sperm function was affected by the storage temperature of the semen prior to its use in IVF.

### 2.6 Statistical analysis

The sperm parameters were log-transformed to estimate normal distribution. Generalized linear regression (package Lme4, R, 4.3.0) was used to test the effect of storage (4 °C or 16–18 °C) against the fresh semen sample, and the effect of storage temperature (4 °C or 16–18 °C) on semen quality parameters (damaged membranes, intact membranes, %DFI, HDS, Live superoxide +, Live superoxide −, Live H_2_O_2_+, Live H_2_O_2_−, Dead superoxide +, Dead H_2_O_2_+, Dead H_2_O_2_−). Treatment and days of storage (6 or 7 days) were included as fixed effects and Boar was included as a random effect to account for the individual variability among boars from which the sperm samples were collected. Model selection was based on Akaike Information Criterion (AIC) and likelihood ratio tests. Based on these criteria, storing the sample 6 or 7 days did not affect treatment outcome and therefore length of storage could be removed from the models. Therefore, the results from day 6 and 7 were combined and presented as one variable (day 7). Furthermore, the results from wells separately, and from wells and drops together were tested and yielded similar results. The results did not differ and therefore all replicates are included here.

The impact of treatment on oocyte developmental competence parameters (Proportion cleaved 48 h after fertilization, Proportion cleaved above 2 cell stage 48 h after fertilization, Proportion embryos day 5 and 6 days after fertilization) were assessed through mixed-effect logistic regression analysis (glmer from the MASS package in R). This analysis utilized a binary distribution, with replicate as a random factor and weighting for group size to determine the odds ratio. The odds ratio (OR) <1 indicates a negative effect of the treatment. To calculate the effect of treatment on the number of spermatozoa attached to the zona pellucida, non-parametric tests (Mann–Whitney) was used (wilcox.test model in R).

Raw *p*-values are presented (no adjustment for multiple testing was included); *p* < 0.05 was considered significant.

## 3 Results

The results for sperm membrane integrity and fragmented DNA are reported in Table 1. Membrane integrity in stored samples was similar to the fresh samples but was different between storage temperatures, being higher in the samples stored at 16 °C than at 4 °C (89 ± 4, 83 ± 0.09, and 91 ± 0.05%, for fresh samples and samples stored at 4 °C and 16 °C, respectively). There were more sperm with damaged membranes in the samples stored at 4 °C than at 16 °C (0.18 ± 0.10 and 0.10 ± 0.05%, respectively).

**Table 1:** Effect of storage on membrane integrity, DNA fragmentation index and High DNA stainability in boar semen stored for 1 week at 4 °C or 16-18 °C (*n* = 9).

Parameter	Fresh	4 °C storage	*p*-value Fresh vs. 4 °C	16 °C storage	*p*-value Fresh vs. 16 °C	*p*-value 4 °C vs. 16-18 °C
Intact membranes (%)	89 ± 4%	83 ± 0.09%	0.10	91 ± 0.05%	NS	0.01
Damaged membranes (%)	0.12 ± 0.05%,	0.18 ± 0.10%	0.07	0.10 ± 0.05%	0.35	0.006
DNA fragmentation (%DFI)	3.58 ± 1.3	3.74 ± 2.25	0.87	7.40 ± 3.36	<0.001	0.0002
HDS (%)	0.27 ± 0.06	0.39 ± 0.18	0.14	0.40 ± 0.20	0.08	0.73

HDS, high DNA stainability.

The %DFI was similar in fresh samples on Day 0 and in cold-stored samples on day 7 but was higher in samples stored at 16 °C (3.58 ± 1.30, 3.74 ± 2.25, and 7.40 ± 3.36% for fresh samples and samples stored at 4 °C and 16-18 °C, respectively; *p* < 0.001). The proportion of sperm with HDS was not different (0.27 ± 0.06, 0.39 ± 0.18, and 0.40 ± 0.02% for fresh samples and samples stored at 4 °C and 16-18 °C, respectively; *p* > 0.05).

The ROS status of the sperm samples is shown in Table 2. The proportion of live superoxide positive sperm was higher in cold-stored samples than in fresh samples (*p* < 0.001), but the proportion of spermatozoa in the other two categories for superoxide were not different. The proportion of live superoxide negative spermatozoa was higher in the fresh samples than for samples stored at 4 °C (82.04 ± 1.07 and 75.83 ± 2.49%, respectively; *p* = 0.04). In addition, although there was no difference between the proportions of live superoxide negative spermatozoa in fresh samples and in samples stored at 16-18 °C, there was a difference between samples stored at 4 °C and 16-18 °C (75.83 ± 2.49 and 83.44 ± 1.56%, respectively; *p* < 0.008). For hydrogen peroxide production, only the proportion of live hydrogen peroxide positive spermatozoa was different, being greater in the stored samples than in the fresh sperm samples (*p* < 0.001). There was no difference between samples stored at 4 °C and 16-18 °C.

**Table 2:** Effect of temperature of storage on reactive oxygen species in boar spermatozoa stored for 7 days at 4 °C or 16 °C (*n* = 9).

	Fresh	4 °C storage	*P*-value Fresh vs. 4 °C	16-18 °C storage	*p*-value Fresh vs. 16-18 °C	*P*-value 4 °C vs. 16-18 °C
Live superoxide positive (%)	5.38 ± 0.87	13.54 ± 6.23	<0.001	6.79 ± 2.37	0.35	<0.0001
Live superoxide negative (%)	82.04 ± 1.07	75.83 ± 2–49	0.04	83.44 ± 1.56	0.69	0.008
Dead superoxide positive (%)	12.04 ± 1.09	9.29 ± 0.80	0.27	9.11 ± 0.29	0.08	0.47
Live hydrogen peroxide positive (%)	0.20 ± 0.13	0.95 ± 1.44	<0.001	0.74 ± 0.43	0.001	0.61
Live hydrogen peroxide negative (%)	87.50 ± 1.12	89.37 ± 0.86	0.25	89.46 ± 1.56	0.23	0.97
Dead hydrogen peroxide positive (%)	0.025 ± 0.007	0.0527 ± 0.02	0.55	0.037 ± 0.008	0.69	0.28
Dead hydrogen peroxide negative (%)	12.27 ± 1.11	9.62 ± 0.76	0.06	9.83 ± 1.04	0.06	0.99

**Figure 1:**
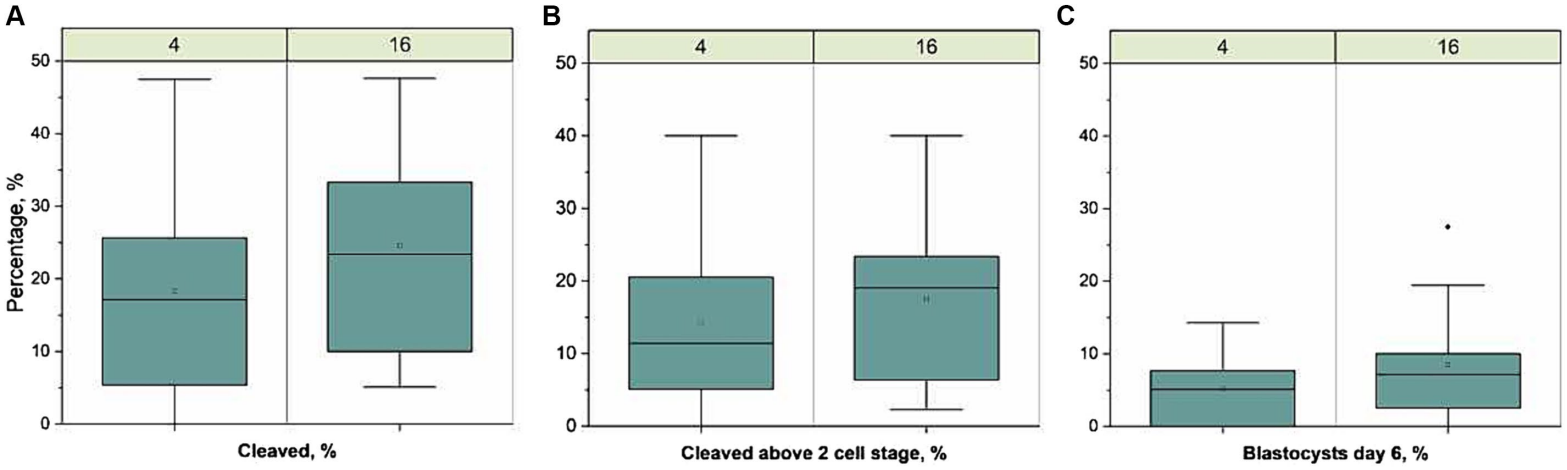
Developmental competence of oocytes fertilized with sperm samples stored for 1 week at 4 °C. (*n* = 436) or 16-18 °C (*n* = 438), run in 11 replicates. Boxplots present the proportion of **(A)** cleaved zygotes 44 h after fertilization from total matured oocytes, **(B)** cleaved above 2 cell stage 44 h after fertilization from total matured oocytes, and **(C)** blastocysts day 6 after fertilization from total matured oocytes,. The line represent the median of all replicates, the open circles represent the mean, the box the interquartile range (IQR), the whiskers length are 1.5 × IQR and filled dot represents outlier (>1.5 × IQR). There was a lower proportion of cleaved zygotes in the 4 °C group compared to the 16-18 °C group (*p* = 0.04) but there was no significant difference in the proportion of zygotes cleaved above the two cell stage or in blastocysts between the two groups.

An overview of the IVF results is presented in Figure 1. The total number of oocytes developing to blastocyst on Day 6 was 39 and 25 for sperm samples stored at 16-18 °C and 4 °C, respectively. The proportion of oocytes developing to blastocyst on Day 6 (mean ± sd) was 9.0 ± 8.0 and 6.0 ± 5.0%, for 16-18 °C and 4 °C, respectively, and was not different between treatments. The proportion cleaved 44 h after fertilization was 25 ± 16 and 20 ± 15% (*p* = 0.04), whereas the proportion cleaved above 2 cell stage was 19 ± 12 and 15 ± 13% for 16-18 °C and 4 °C, respectively. There was no difference between the proportions cleaved above 2 cell stage. The number of spermatozoa attached to the zona pellucida [median (min-max)] in the 16-18 °C group, i.e., 5 (0–55) was not different from the 4 °C group, i.e., 3 (0–13) (*p* = 0.36).

## 4 Discussion

The purpose of this experiment was to evaluate sperm quality and fertilizing capability of boar sperm samples after storage in AndroStar Premium for 6 or 7 days at 4 °C compared to conventional storage at 16–18 °C. The sperm samples were prepared for IVF using a low density colloid to separate the spermatozoa from extender and seminal plasma, i.e., to avoid selecting only the spermatozoa most capable of fertilization, which would have been the case if a high density colloid was used (22). Such selection would tend to negate temperature-induced differences in fertilizing capacity, since only a few spermatozoa are required to achieve fertilization. Thus, although it should be possible to detect differences in fertilizing capacity due to sperm storage, if they exist, no such differences were apparent here.

The results showed that most aspects of sperm quality were not different between the two storage temperatures, with the exception of %DFI, which was lower for the samples stored at 4 °C, and the proportion of live superoxide positive spermatozoa, which was higher for samples stored at 4 °C. Although there was a small difference between the number of zygotes at the two cell stage, the number developing beyond this stage was not different between the two groups. These results are interesting since they indicate that boar spermatozoa can be cooled to 4 °C in AndroStar Premium, and stored at this temperature, without suffering from irreversible functional damage, i.e., cold-shock.

A previous report on cold-storage of boar semen in BTS showed that sperm cooled to 10 °C or 5 °C suffered from cold shock, with a loss of motility and viability, with a sub-lethal imbalance of ATP among the surviving sperm population if stored for more than 120 h (16). In contrast, in our study, not only was sperm viability (membrane integrity) retained in AndroStar Premium at 4 °C, but fertilizing ability in IVF was not different to the conventionally stored samples. Waberski et al. (18) reported that boar sperm viability was maintained in AndroStar^®^ Premium at low temperatures, as opposed to Beltsville Thawing Solution, while Jäkel et al. (23) observed that there was no difference in pregnancy results following AI with semen samples stored at 5 °C compared to 17 °C. On the other hand, Menezes et al. (17) reported that although membrane integrity was maintained in boar semen stored at 5 °C, sperm motility was lower than in samples stored at 17 °C. These results are similar to those for membrane integrity in the present study. However, it is possible that spermatozoa stored at low temperatures require a longer incubation time prior to motility analysis to regain their full motility. This possibility was not examined here. According to the manufacturer, the extender contains membrane stabilizers and capacitation inhibitors, which help to maintain sperm quality long-term (24); it is possible that these inhibitors also reduce sperm motility.

Chromatin integrity was not analysed in the previous studies on cold storage of boar semen. This is surprising since the proportion of boar sperm with single-stranded DNA breaks was observed to rise after 2–3 days of storage at 16–18 °C (13–15), suggesting that this is a critical point for boar sperm storage. Since standard praxis is to store boar semen for up to 5 days before use, samples stored for more than 3 days may potentially have increased proportions of spermatozoa with fragmented chromatin. Spermatozoa with damaged chromatin are capable of fertilization, and the DNA-repair mechanisms of the oocyte can correct some damage (25). If the damage is too great, embryonic development is halted at some stage, potentially being seen as a reduction in litter size, or even a failed pregnancy if too few embryos survive to implant (26). Litter size was shown to be negatively correlated with DNA fragmentation, at least in Norwegian Landrace and Duroc breeds (27). In the present study, the %DFI was lower in the samples stored at 4 °C than in the samples stored at 16–18 °C, which is very promising for avoiding storage-induced DNA damage.

Reactive oxygen species, such as superoxide and hydrogen peroxide, are produced as byproducts of metabolism (28) during glycolysis or oxidative phosphorylation. Previously it was thought that different animal species tend to use mainly one or the other route for sperm ATP-production, but recently it was suggested that spermatozoa may be able to switch from one to the other according to their immediate environmental conditions (29). Regardless of how superoxide is produced, it is assumed that superoxide is converted to hydrogen peroxide by superoxide dismutase (30). Physiological concentrations of ROS are required for normal sperm capacitation (31) but excessive concentrations cause loss of sperm function. Lipid peroxidation and loss of motility were induced in boar spermatozoa by hydrogen peroxide (32), whereas acrosome exocytosis and glycolysis were observed to be caused by hydrogen peroxide (33). Our results showed that there were differences in ROS production in stored boar spermatozoa according to storage conditions, with more superoxide production in the samples stored at 4 °C than in conventionally stored samples. Moreover, more hydrogen peroxide was produced in the stored samples than in the fresh samples. Although membrane integrity was maintained better in the sperm samples stored at 16–18 °C than at 4 °C, DNA fragmentation was actually lower in the samples stored at 4 °C than at 16–18 °C. Thus, either the semen extender contained sufficient antioxidants to deactivate the ROS produced (34), or ROS were not responsible for membrane damage and DNA damage in boar sperm stored under the conditions of this study. These results are interesting and warrant further investigation. Previous studies with stallion sperm also revealed a situation in which increased superoxide production was not associated with declining sperm quality (35), and increased superoxide production was not linked to increased hydrogen peroxide production. Furthermore, another study revealed that hydrogen peroxide and superoxide are present in different compartments of stallion spermatozoa (36).

Our results show that boar semen can be stored refrigerated without losing its fertilizing capacity, at least in IVF, if it is extended in AndroStar Premium. However, the fertility of boar semen stored in this manner was not tested in an AI trial in this study. Previous studies have used cooled semen for AI after storage for 1 or 2 days, but to our knowledge, no studies have been done on fertility after 7 days of storage. Jäkel et al. (23) did not detect a difference in fertility when AI was performed with samples stored for 72 h at 5 °C and 17 °C.

The possibility of cooling boar semen for refrigerated storage and transport could help to avoid loss of quality during transport in the summer months. Although the infrastructure on pig farms is set up for storage of boar semen at 16–18 °C, it would not be too difficult to introduce refrigerated storage since most farms are already equipped with a refrigerator for storing medication and other perishable items. Furthermore, refrigerated transport is commonly used to transport food supplies all over the world. Therefore, refrigerated boar semen could offer considerable advantages over conventional storage temperatures for pig breeding, particularly in view of predicted climate changes.

## 5 Conclusion

The fertility of boar semen doses stored in AndroStar Premium at 16–18 °C or 4 °C for up to 7 days was not different in terms of number of blastocysts developing after *in vitro* fertilization. Sperm quality was affected slightly, in that membrane integrity was better in samples stored at 16–18 °C, but DNA fragmentation was less in the samples stored at 4 °C. Therefore, cold storage of boar semen is possible without a detrimental effect on fertility. It would be a viable alternative to current praxis for pig producers, allowing better control of semen doses during transport in the summer months.

## Data Availability

The original contributions presented in the study are included in the article/supplementary material, further inquiries can be directed to the corresponding author.
